# Long non-coding RNA TUG1 is involved in cell growth and chemoresistance of small cell lung cancer by regulating LIMK2b via EZH2

**DOI:** 10.1186/s12943-016-0575-6

**Published:** 2017-01-09

**Authors:** Yuchun Niu, Feng Ma, Weimei Huang, Shun Fang, Man Li, Ting Wei, Linlang Guo

**Affiliations:** 1 Department of Pathology Zhujiang Hospital, Southern Medical University, 253 Gongye Road, Guangzhou, 510282 People’s Republic of China; 2 Department of Oncology, The First Affiliated Hospital of Hebei North University, Zhangjiakou, China; 3 Department of Oncology, Zhujiang Hospital, Southern Medical University, Guangzhou, China

**Keywords:** TUG1, Small cell lung cancer (SCLC), Cell growth, Chemoresistance

## Abstract

**Background:**

Taurine upregulated gene1 (TUG1) as a 7.1-kb lncRNA, has been shown to play an oncogenic role in various cancers. However, the biological functions of lncRNA TUG1 in small cell lung cancer (SCLC) remain unknown. The aim of this study is to explore the roles of TUG1 in cell growth and chemoresistance of SCLC and its possible molecular mechanism.

**Methods:**

The expression of TUG1 in thirty-three cases of SCLC tissues and SCLC cell line were examined by quantitative RT-PCR (qRT-PCR). The functional roles of TUG1 in SCLC were demonstrated by CCK8 assay, colony formation assay, wound healing assay and transwell assay, flow cytometry analysis and in vivo study through siRNA or shRNA mediated knockdown. Western blot assays were used to evaluate gene and protein expression in cell lines. Chromatin immunoprecipitation (ChIP) and RNA binding protein immunoprecipitation (RIP) were performed to confirm the molecular mechanism of TUG1 involved in cell growth and chemoresistance of small cell lung cancer.

**Results:**

We found that TUG1 was overexpressed in SCLC tissues, and its expression was correlated with the clinical stage and the shorter survival time of SCLC patients. Moreover, downregulation of TUG1 expression could impair cell proliferation and increased cell sensitivity to anticancer drugs both in vitro and in vivo. We also discovered that TUG1 knockdown significantly promoted cell apoptosis and cell cycle arrest, and inhibited cell migration and invasion in vitro . We further demonstrated that TUG1 can regulate the expression of LIMK2b (a splice variant of LIM-kinase 2) via binding with enhancer of zeste homolog 2 (EZH2), and then promoted cell growth and chemoresistance of SCLC.

**Conclusions:**

Together, these results suggested that TUG1 mediates cell growth and chemoresistance of SCLC by regulating LIMK2b via EZH2.

**Electronic supplementary material:**

The online version of this article (doi:10.1186/s12943-016-0575-6) contains supplementary material, which is available to authorized users.

## Background

Small-cell lung cancer (SCLC) is a highly lethal malignancy that accounts for 10–15% of lung cancers [1]. SCLC is characterized by a rapid doubling time, high growth fraction, and early development of widespread metastases [2]. Although the incidence of SCLC is reportedly decreasing over time, 5-year survival rates is still lower than 10%[3]. SCLC is highly sensitive to initial chemotherapy and radiotherapy; however, most patients eventually die of widespread metastasis and rapid development of chemoresistance to chemotherapy [4, 5]. In addition, though genetic changes have been reported in SCLC [6], the precise molecular mechanisms involved in SCLC development and chemoresistance remain to be fully elucidated.

Recently, research has postulated a class of non-protein-coding RNAs (ncRNAs) that longer than 200 nucleotides in length, defined as long non-coding RNAs (lncRNAs), participates in cell biological processes and human disease pathogenesis [7, 8]. lncRNAs are poorly conserved and regulate gene expression at various levels, such as chromatin modification, transcription and post-transcriptional processing [9, 10]. With more and more studies on lncRNA, some researchers classify lncRNA for five broad categories: sense, antisense, bidirectional, intronic, intergenic; and summarize four known molecular functions of lncRNAs: signal, decoy, guide, and scaffold [11]. Interestingly, increasing evidence suggests that lncRNAs play a important role in tumorigenesis, and their aberrant expression confers tumor initiation, cancer cell growth and apoptosis, chemoresistance, invasion and metastasis [12–14]. For example, promotion of lung cancer metastasis by lncRNA MALAT1 (Metastasis Associated Lung Adenocarcinoma Transcript 1); control of hepatocellular cancer cell growth and apoptosis by MEG3; regulation of oesophageal adenocarcinoma cell proliferation and migration by HNF1A-AS1 [15–17]. In addition, studies showed that the long non-coding RNA HOTTIP promotes gemcitabine resistance by regulating HOXA13 in pancreatic cancer [14]. Our laboratory also reported that lncRNA HOTAIR affects chemoresistance by regulating HOXA1 methylation in SCLC [18]. However, functional roles of lncRNAs in SCLC have not been well documented.

The TUG1 (Taurine upregulated gene) lncRNA, located at chromosome 22q12, was originally identified as a transcript up-regulated by taurine [19]. Recently, accumulating evidence has shown that TUG1 is a negative prognostic factor for osteosarcoma patient survival, and high expression of TUG1 in patients has been correlated with enhanced bladder and esophagus cancer cells proliferation and metastasis [20–22]. In previous study, researcher found TUG1 could induced by p53, then binds to PRC2, and play a key role in cell-cycle regulation [23]. Some studies have explored that TUG1 may regulate genes expression through binding to PRC2. For instance, TUG1 could regulate the expression of HOXB7 by binding to PRC2, then affects cell proliferation in human non-small cell lung cancer [24]. In gastric cancer, TUG1 epigenetically silencing of p57 by binding with PRC2 to regulates cell proliferation [25]. However, little is known about TUG1 in SCLC.

In this study, we attempted to explore the potential involvement of TUG1 in SCLC. We found that TUG1 was upregulated in SCLC tissues than matched adjacent normal tissues and its upregulation is related with poor prognosis. Knockdown of TUG1 impairs proliferation, migration, invasion and induces cell apoptosis and cell-cycle arrest of human SCLC cell lines. Moreover, we identify the role of TUG1 in chemoresistance in SCLC cells for the first time. Additionally, we found TUG1 affect cell growth and chemoresistance by regulating LIMK2b expression via binding with EZH2. Taken together, our findings suggest that TUG1 may be a novel potential molecular target for treating SCLC patients.

## Results

### The expression of TUG1 increased in SCLC tissues and was associated with clinical stage and survival

**Fig. 1:**
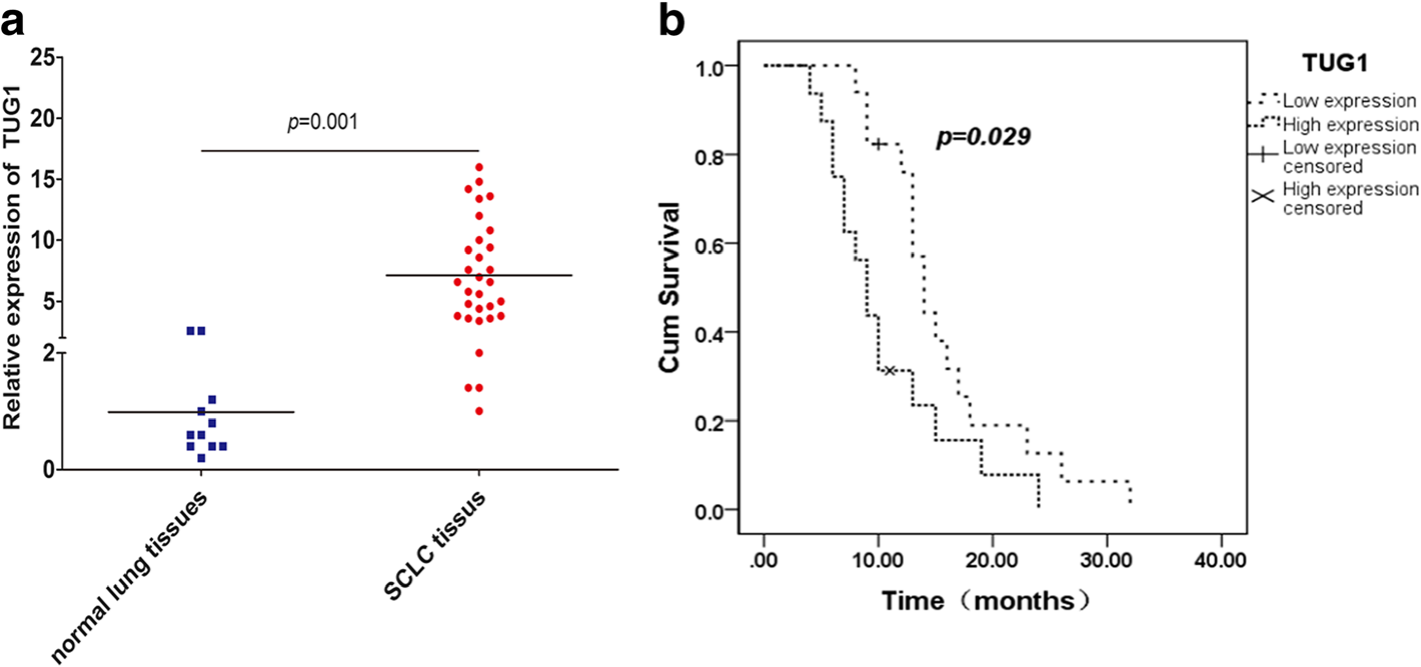
Relative TUG1 expression and its clinical significance in SCLC tissues. **a** The expression levels of TUG1 in SCLC tissues (*n* = 33) and adjacent non-tumor tissues (*n* = 11). **b** Kaplan–Meier analysis of overall survival of 33 patients with SCLC based on TUG1 expression

To investigate the clinicopathological features of TUG1 expression in SCLC, qRT-PCR was performed in 33 tumor samples from SCLC patients. TUG1 expression level was significantly higher in SCLC tumor tissues than those in normal counterparts (P < 0.01) (Fig. 1a). Table 1 summarizes the correlation between TUG1 expression and clinicopathological parameters of SCLC patients. The data indicated that higher expression of TUG1 in extensive disease-SCLC (ED-SCLC) than in limited disease SCLC (LD-SCLC) (P = 0.011). Specifically, we observed higher expression of TUG1 in smoking patients. Kaplan-meier survival analysis based on TUG1 expression showed that high TUG1 expression was correlated with poorer patient survival (Fig. 1b). However, no significant difference was observed with respect to sex (female and male) and age (≤62 years and >62 years) in our study. Taken together, these results indicated that TUG1 overexpression in SCLC tissues was correlated with stage and survival of SCLC patients.

**Table 1:** Association of TUG1 expression and clinicopathological characteristics in 33 SCLC patients

Characteristics	Total (*n* = 33)	TUG1 expression		*χ* ^2^	*P* value*
		Low expression	High expression		
Gender				1.816	0.174
Male	26	15 (57.7)	11 (42.3)		
Female	7	2 (50.0)	5 (50.0)		
Age (years)				0.299	0.420
≤62 year	19	9 (47.4)	10 (52.6)		
>62 year	14	8 (57.1)	6 (42.9)		
Smoking history				3.529	0.032
YES	20	7 (35.0)	13 (65.0)		
NO	13	10 (76.9)	3 (23.08)		
Disease stage				6.651	0.011
Limited disease (LD)	16	12 (75.0)	4 (25.0)		
Extensive-stage disease (ED)	17	5 (29.4)	12 (70.6)		
Status				4.520	0.036
Survival	10	8 (80.0)	2 (20.0)		
Death	23	9 (39.1)	14 (60.9)		

*For analysis of correlation between of TUG1 expression levels and clinical features, Fisher’s Exact Test were used. Results were considered statistically significant at *P* <0 .05

### TUG1 was upregulated in SCLC cell lines and affected cell proliferation in vitro and in vivo

**Fig. 2:**
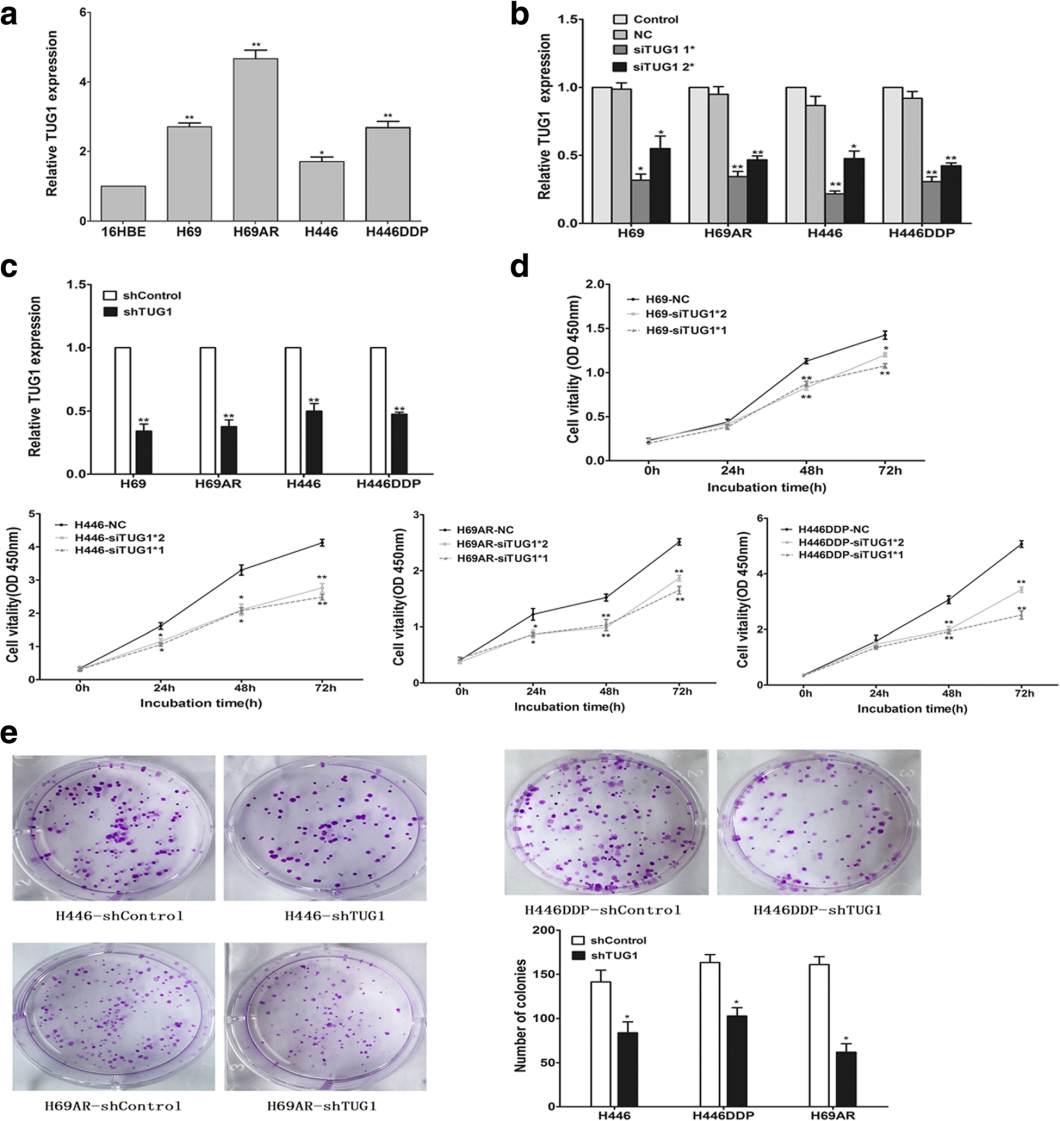
TUG1 was up-regulated in SCLC cell lines and TUG1 knockdown inhibited cell proliferation in vitro. **a** The expression of TUG1 was assessed in SCLC cell lines compared with the normal bronchial epithelial cell line (16HBE) by qRT-PCR. **b** **c** Inhibition of TUG1 by transfection of TUG1 siRNAs or sh RNA in H69、H69AR、H446、H446DDP cells. **d** CCK-8 proliferation assays were used to determine the cell viability for siTUG1 transfected SCLC cells. Experiments were performed in triplicate. **e** Colony formation assays were performed to determine the proliferation of shTUG1 transfected H446, H446DDP and H69AR cells. Representative photographs are shown, and the numbers of colonies were counted. *, *P* < 0.05; **, *P* < 0.001

To further investigate the role of TUG1 in SCLC cells, we evaluated the expression of TUG1 in SCLC cell lines (H69, H69AR, H446, H446DDP) and in the normal bronchial epithelial cell line (16HBE) by qRT- PCR. As shown in Fig. 2a, all SCLC cell lines expressed high levels of TUG1 compared with 16HBE. We then tested whether TUG1 was functionally involved in SCLC cell growth. We first designed three different TUG1 siRNAs to transfect these four cell lines. qRT-PCR analysis was conducted at 24 h post-transfection and showed that siTUG1 1* and siTUG1 2* had higher efficiency of interference than siTUG1 3* (Additional file 1: Figure S1 A-D). Then we chose siTUG1 1* and siTUG1 2* for the following experiments (Fig. 2b). Moreover, we also established stable TUG1 knockdown SCLC cell lines by retrovirus infection (Fig. 2c). CCK-8 assay and colony formation assay were used to detect the effect of TUG1 knockdown on growth of the SCLC cell lines. As shown in Fig. 2d, SCLC cells transfected with siTUG1 showed greatly reduced cell proliferation rate. Similarly, the colony formation assay demonstrated that the number of colonies decreased significantly in SCLC cells transfected with shTUG1 as compared with shControl (Fig. 2e).

**Fig. 3:**
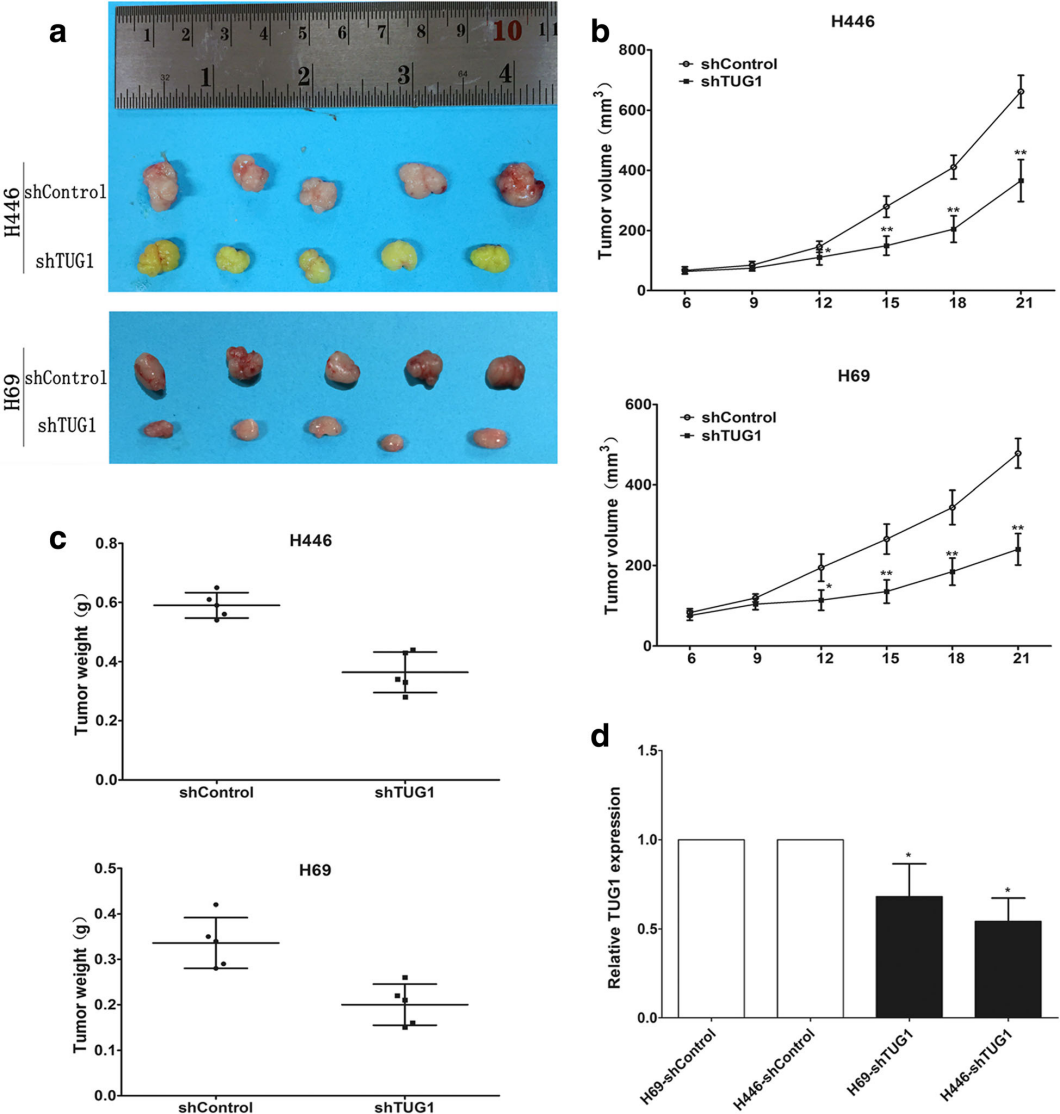
Effects of TUG1 knockdown on tumor growth in vivo. **a** Tumors formation of cells stably with lowTUG1 expression (*N* = 5 mice for each group). **b** Growth curve of tumor volumes. **c** Tumor weights were determined. **d** qRT-PCR was conducted to detect the expression of TUG1. *, *P* < 0.05; **, *P* < 0.001

The tumorigenic properties of TUG1 in vivo were performed in male nude mouse xenograft. We injected H446 and H69 cells transfected with either shControl or shTUG1 into nude mice. As shown in Fig. 3a-c, downregulation of TUG1 significantly inhibited tumor growth. We also performed qRT-PCR to detect the expression of TUG1 in tumor tissues selected from mice (Fig. 3d). Results showed that the expression levels of TUG1 in the shTUG1 group were lower than those in the control group. Taken together, these data suggested the TUG1 affects SCLC cell proliferation and growth.

### TUG1 involved in SCLC cell migration and invasion

Wound healing assay and transwell assay were performed to investigate whether TUG1 had a functional role in cell migration and invasion in SCLC. Result of wound healing assay demonstrated the migration distance of SCLC cells infected by shTUG1 was significantly wider than the shControl group (Additional file 2: Figure S2 A). In the transwell assay, the number of shTUG1 infected cells that migrated through the membrane were significantly less than the shControl group (Additional file 2: Figure S2 B). These results showed that inhibition of TUG1 could significantly impair SCLC cell migration and invasion ability compared with control group.

### TUG1 regulated cell apoptosis and cell cycle in SCLC cells

To further probe the potential mechanisms underlying the growth-inhibitory effects of TUG1 knockdown, we assessed cell apoptosis and cell-cycle in H446 and H69 cells. Flow cytometry analysis demonstrated that TUG1 knockdown led to a significant increase of cell apoptosis (P < 0.05) and a significant accumulation of cells at G1-phase (P < 0.001, Additional file 3: Figure S3 A and B). These data suggested that TUG1 mediated promotion of SCLC cell growth may be mediated by regulation of the apoptosis and G1-phase . Moreover, to investigate the possible mechanisms of TUG1 in chemoresistance, we also conducted flow cytometry analysis to examine the impact of TUG1 on apoptosis when exposed to chemotherapeutic drugs. The results showed that downregulation of TUG1 in H446DDP and H69AR cells resulted in increased drug-induced apoptosis after treatment with ADM, DDP or VP-16 (Additional file 4: Figure S4).

### TUG1 expression was associated with SCLC chemosensitivity in vitro and in vivo

**Fig. 4:**
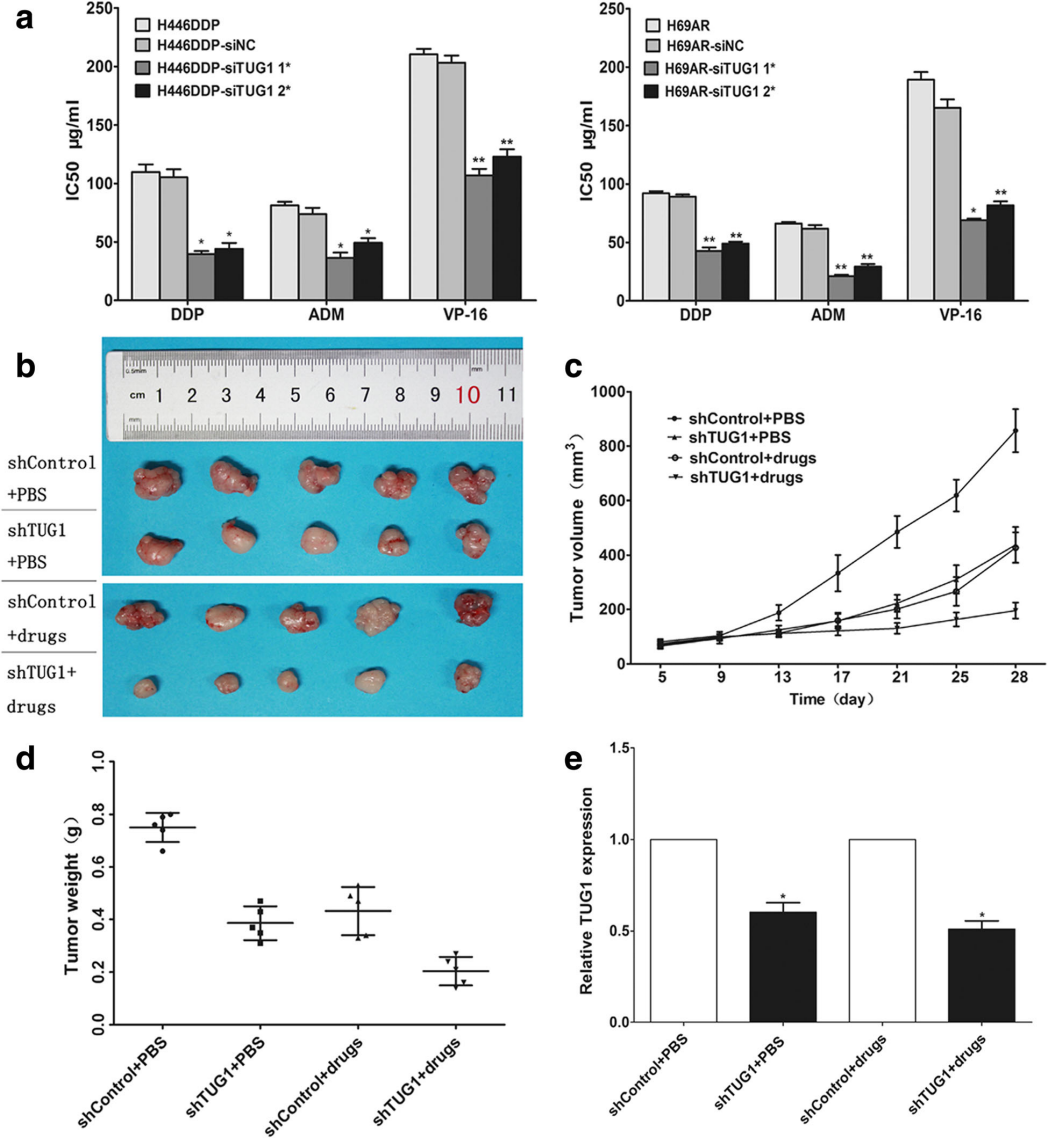
Knockdown TUG1 enhanced the chemosensitivity of SCLC cells to anticancer drugs both in vitro and in vivo. **a** The sensitivities of cells to chemotherapy drugs (CDDP, ADM or VP-16) were measured after H69AR and H446DDP cells transfected with siTUG1 by CCK-8 assay. **b** Tumors from all mice in each group (Each group has five mice). H446DDP cells were transduced with shControl or shTUG1 as indicated. After cells (3x10^7^) were injected into mice, chemotherapeutics or PBS were injected intraperitoneally as indicated. **c** Growth curve of tumor volumes. **d** Tumor weights were determined. **e** qRT-PCR was conducted to detect the average expression of TUG1. *, *P* < 0.05; **, *P* < 0.001

To further investigate the impact of TUG1 on SCLC chemosensitivity, we detected the differential expression of TUG1 in SCLC drug-sensitive cells (H69 and H446) and drug-resistant cells (H69AR andH446DDP) by qRT-PCR. The results showed that TUG1 over-expression in H69AR and H446DDP cells than that in H69 and H446 cells (Fig. 2a). After knockdown of TUG1, the IC50 values of H446DDP and H69AR cells significantly decreased with treatment of chemotherapeutic drugs including DDP, ADM or VP-16 (Fig. 4a).

We then used a nude mouse xenograft model to further investigate the ability of TUG1 to confer chemoresistance in SCLC. H446DDP cells transfected with shTUG1 or shControl were subcutaneously injected into mice. As shown in Fig. 4b, tumor growth was inhibited in the shTUG1 group treated with PBS or drugs (DDP and VP-16) compared with the controls. Tumor grew significantly more slowly in mice following combined drugs treatment and TUG1 knockdown. Four weeks later, the mean tumor volume for the TUG1-knockdown group and the drugs group was obviously smaller than that of the control group (Fig. 4c). Moreover, combined treatment with TUG1 knockdown and drugs led to an even further reduction in tumor volume. Similarly, the average tumor weight in shTUG1 group combined treatment with drugs showed a similar trend (Fig. 4d). qRT-PCR analysis of TUG1 expression found it to be significantly lower in tumor tissues formed from shTUG1 group than those from controls (Fig. 4e). These results suggested that downregulation of TUG1 increased the in vivo chemosensitivity of H446DDP cells to drugs.

### TUG1 affected cell growth and chemoresistance of SCLC by regulating LIMK2b expression

**Fig. 5:**
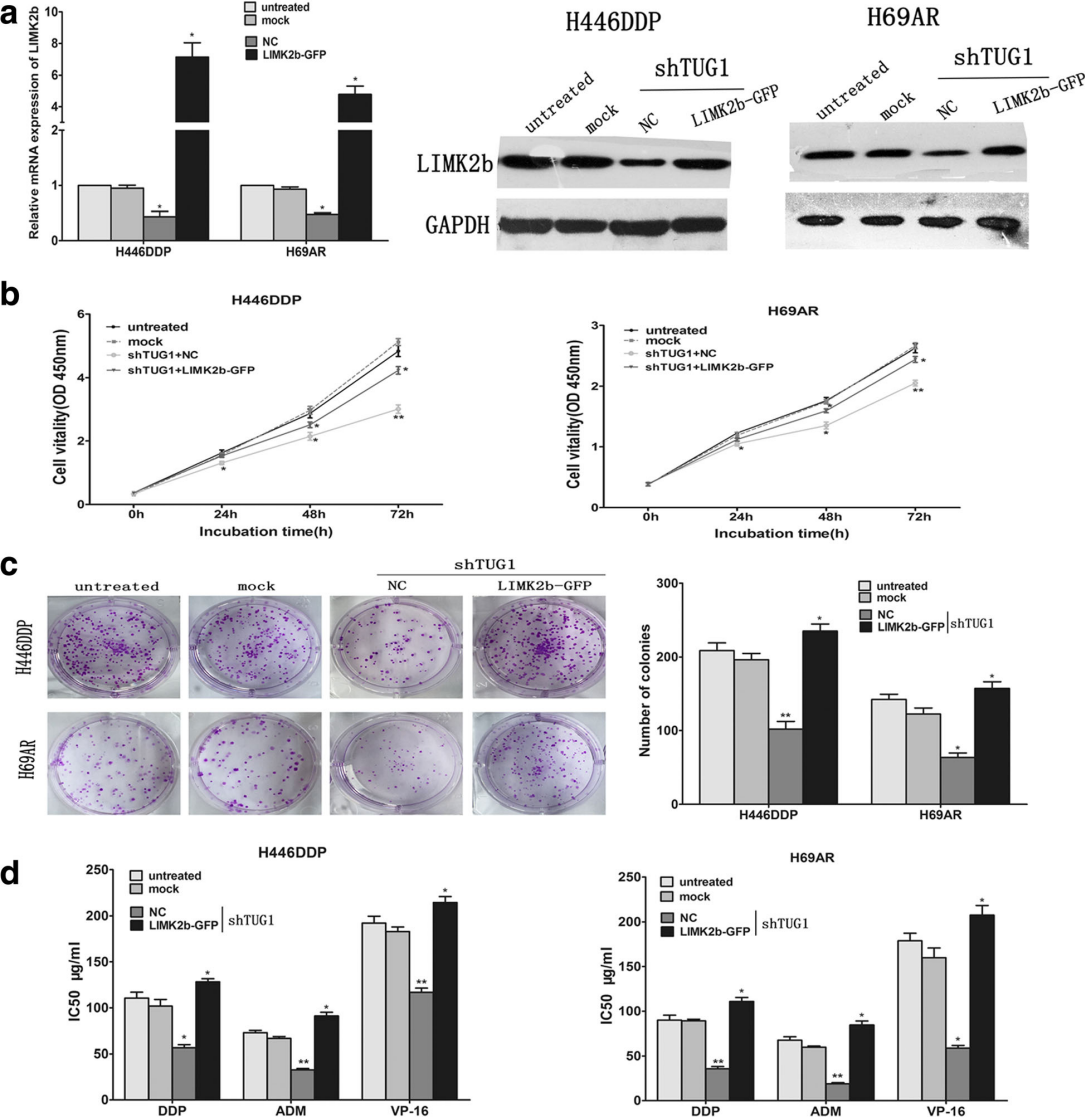
TUG1 affected cell growth and chemoresistance of small cell lung cancer by regulating LIMK2b. **a** qRT-PCR and western blot analysis for LIMK2b in H446DDP-shTUG1 and H69AR-shTUG1 cells transfected with LIMK2b-GFP (NC, LIMK2b-GFP). **b** CCK-8 proliferation assay were used to determine the cell viability (knockdown TUG1 while overexpression LIMK2b). **c** Representative images of Colony formation assays for proliferative cells knockdown TUG1 while overexpression LIMK2b. **d** Survival of H446DDP and H69AR cells transfected with shTUG1 while overexpression LIMK2b significantly increased compared with those transfected with negative control or mock transfected after treatment of CDDP, ADM or VP-16

To determine how TUG1 affects cell growth and chemoresistance of SCLC, we conducted the bioinformatics analysis to identify its potential downstream target genes. We first searched the database and found that TUG1 was previously identified to have an enhancer-like function and positive influence on the neighboring protein-coding genes [26]. We then predicted 12 TUG1 nearby coding genes (distance <300 kb) including LIMK2b by searching UCSC Genome database (http://genome.ucsc.edu/). Finally, we confirmed LIMK2b may be the potential target gene in SCLC according to our cDNA microarray analysis in which 1.5-fold upregulation in drug resistance SCLC cells as compared to the parental SCLC cells. We further performed qRT-PCR and Western blot analysis to confirm our supposition. The results showed that knockdown of TUG1 can inhibit LIMK2b expressions both at mRNA and protein levels in H446DDP and H69AR cells (Fig. 5a).

We next examined whether LIMK2b played an important role in TUG1 mediated cell growth and chemoresistance by rescue experiments. Transfection of LIMK2b-GFP in H446DDP and H69AR cells completely reversed the down-regulation of LIMK2b induced by TUG1 (Fig. 5a). CCK8 and colony formation assay results suggested that cotransfection can partially rescue shTUG1 impaired proliferation and chemoresistance (Fig. 5c and d). These results indicate that TUG1 promotes SCLC cell proliferation and chemoresistance partly through downregulation of LIMK2b expression.

**Fig. 6:**
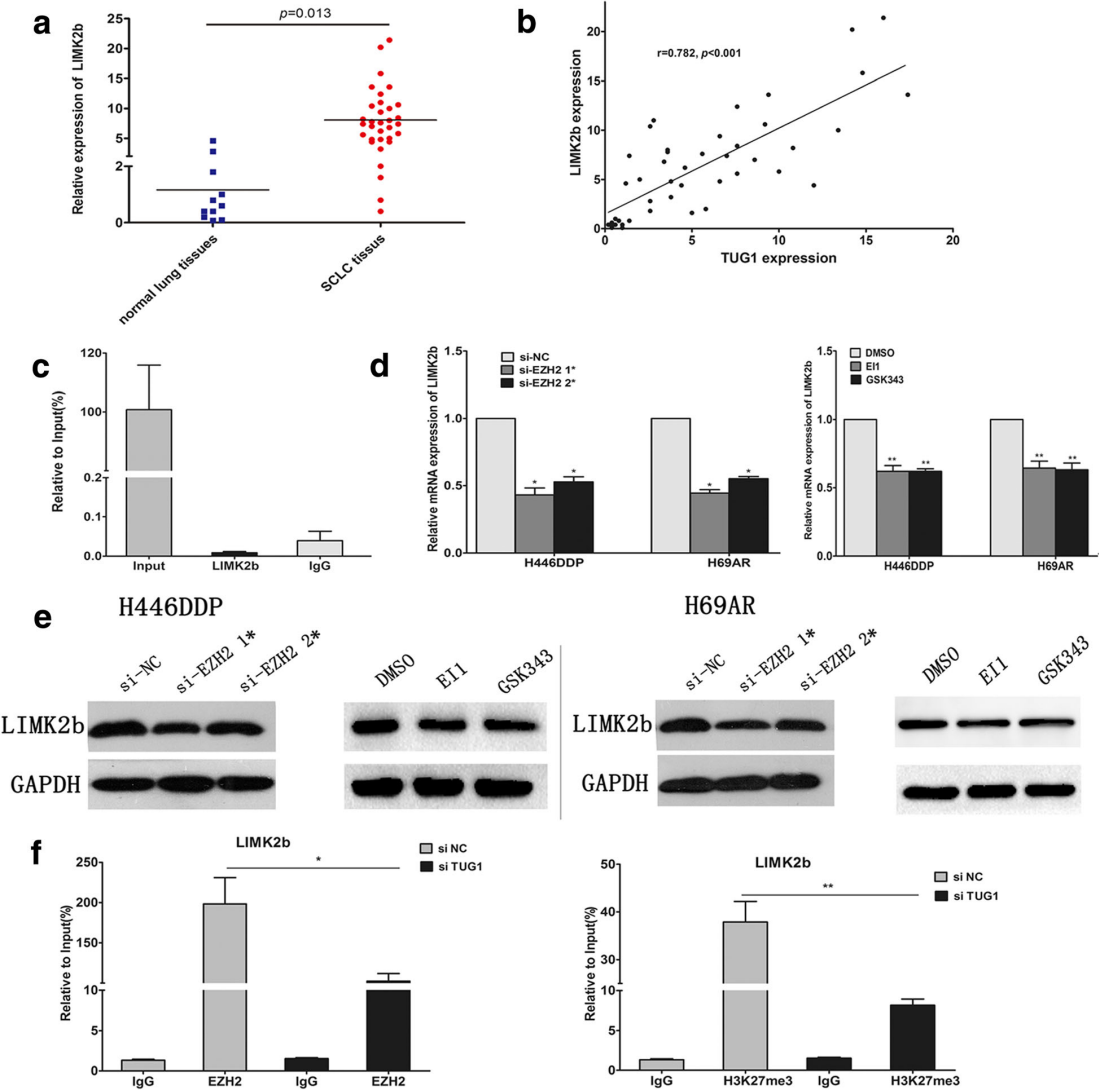
TUG1 regulated LIMK2b expression by binding with EZH2. **a** Expression of LIMK2b in SCLC FFPE tissues by qRT-PCR. **b** The correlation of LIMK2b and TUG1 expression in FFPE tissues. **c** RIP experiments were performed in H69AR cells and the coprecipitated RNA was subjected to qRT-PCR for TUG1. The fold enrichment of TUG1 in LIMK2b RIP is relative to its matching IgG control. **d** **e** qRT-PCR and western blot analysis for LIMK2b in H446DDP, H69AR cells inhibited EZH2 with EI1 or GSK343 or transfected with siRNA targeting EZH2 (si-NC, si-EZH2 1* and si-EZH2 2*). **f** ChIp-qPCR of EZH2 occupancy and H3K27me3 binding in the promoters of LIMK2b in H69AR cells treated with siNC or si-TUG1; IgG as a negative control. *P < 0.05, **P < 0.001

Subsequently, we detected the relationship of TUG1 and LIMK2b in SCLC FFPE tissues. Consistent with the results obtained from SCLC cell lines, LIMK2b was overexpressed in SCLC tissues and the expression levels of LIMK2b were positively correlated with those of TUG1 by qRT-PCR in 33 SCLC FFPE tissues (Fig. 6a and b).

### TUG1 regulated LIMK2b expression by binding with EZH2

On the basis of the study above, we found that TUG1 could regulate the expression of LIMK2b, then we test whether TUG1 has a direct combination with LIMK2b. However, the result of RIP analysis showed no direct combination between TUG1 and LIMK2b (Fig. 6c). TUG1 was previously reported to mediated transcriptional regulation through binding with EZH2 [23–25, 27]. Then we suppose that TUG1 may regulate LIMK2b expression by binding with EZH2. We designed three different EZH2 siRNAs and to transfect H446DDP and H69AR cells (Additional file 1: Figure S1 E-F). To further confirm the results, we also applied EZH2 inhibitors EI1 or GSK343 to treat H446DDP and H69AR cells. The results showed that LIMK2b level was downregulated after EZH2 expressions were inhibited (Fig. 6d and e).

Furthermore, we conducted ChIP assays and found that knockdown of TUG1 decreased the binding of EZH2 and H3K27me3 levels across the LIMK2b promoter compared to cells transfected with siNC (Fig. 6f). Taken together, these data suggest that TUG1 is required to target EZH2 occupancy and activity to epigenetically modulate the expression of LIMK2b.

## Discussion

Recently, the study about the biological function of TUG1 has become one of the hottest topics in various cancer. TUG1 was overexpressed in various solid tumor including osteosarcoma, bladder, esophagus, gastric and liver cancer [20–22, 25, 27]. Nonetheless, the clinical features of TUG1 expression in SCLC have not been reported yet. In this study, we analyzed the expression of TUG1 in 33 cases of human SCLC tissues and found that the high expression level of TUG1 indicates shorter survival time of the SCLC patients. Therefore, our research may provide an independent prognostic factor for SCLC patients.

To explore the functional role of TUG1 in SCLC, we therefore established stable TUG1-downexpressed cells in the study. Our data indicate that TUG1 was upregulated in SCLC, inhibition of TUG1 expression resulted in decreased cell growth and enhanced chemosensitivity both in vitro and in vivo. Moreover, we found that knockdown of TUG1 also increased cell apoptosis, G1 cell-cycle arrest, and impaired SCLC cell migration and invasion ability.

To further investigate the mechanisms of TUG1 involved in cell growth and chemoresistance, we conducted the bioinformatics analysis and found LIMK2b, which located at 300kp of TUG1. LIMK2 is a member of LIM kinase (LIMK) family that includes LIMK1. LIMK2 encodes a kinase that regulates actin dynamics through phosphorylation of cofilin, and comprises two alternative transcripts, LIMK2a and LIMK2b [28–30]. Recent reports illustrate that LIMK2 is involved in tumor growth, and induces migration and invasion of tumor cells [31–33]. Additionally, studies also showed a negative correlation between LIMK2 and anticancer drugs, which suggesting that LIMK2 may be a predictive marker of drug resistance [34, 35]. Previous study showed that LIMK2b could encode only one and a half LIM domains after the first LIM domain partially replaced, which is special to the LIMK2 gene and conserved between murine and human genes [36, 37]. Recent studies demonstrated that LIMK2b is a direct target of p53 and involved in the control of cell proliferation and cell division. The report also showed that LIMK2b has a critical role in promoting the G2/M DNA-damage checkpoint [38]. In the present study, we firstly hypothesized that TUG1 may associated with LIMK2b. Our results showed that knockdown of TUG1 significantly decrease the expression of LIMK2b. Furthermore, the impacts of TUG1 on cell growth and chemoresistance were reversed by concomitant LIMK2b -GFP, which indicating that the effect of TUG1 on cell growth and chemoresistance is partly mediated through LIMK2b. Moreover, we also found that LIMK2b was over- expressed and positively correlated with TUG1 in SCLC tissues. However, RIP analysis indicated that there was no direct combination between TUG1 and LIMK2b, which suggested that TUG1 affected cell growth and chemoresistance is not directly through LIMK2b. The results stated above raises an interesting question: What is the linker between TUG1 and LIMK2b? Several recent studies indicated that TUG1 regulate genes expression by binding with EZH2 to affect cell proliferation in human non-small cell lung cancer, gastric cancer and hepatocellular carcinoma [24, 25, 27]. So, we hypothesized that EZH2 may be a linker between TUG1 and LIMK2b. To prove our hypothesis, we used ChIP assays to demonstrate that knockdown of TUG1 decreased the binding of EZH2 and H3K27me3 levels across the LIMK2b promoter. EZH2 as an important component of polycomb repressive complex2 (PRC2) has been reported to be necessary for the formation of the H3K27me3, and recruits histone deacetylases then resulting in gene transcriptional repression in cancer cells [39–41]. These results suggested that TUG1 epigenetically regulated LIMK2b through EZH2.

## Conclusions

In summary, our study showed that TUG1 was upregulated in SCLC tissues and its overexpression is closely associated with clinical stage and overall survival in patients with SCLC. Furthermore, the effects of TUG1 on cell proliferation, cell apoptosis, cell cycle regulation, migration, invasion and chemoresistance indicated that TUG1 promotes tumorigenesis. We also demonstrated that TUG1 is involved in cell growth and chemoresistance of SCLC through regulating LIMK2b by binding with EZH2. This study may provide a strategy and lead to the development of lncRNAs directed diagnostics and therapeutics against SCLC.

## Methods

### Patients and tissue samples

A total of 33 formalin-fixed, paraffin-embedded (FFPE) tissues (33 primary cancerous, 11 adjacent non cancerous tissues) were collected from patients who had underwent bronchofiberscopy or biopsy for SCLC between the period 2009.1 and 2013.11 and receiving care and follow-up at The First Affiliated Hospital of Hebei North University, informed consent was obtained from all patients before sample collection. The experiments were approved by the Ethics Committee of The First Affiliated Hospital of Hebei North University, and conformed to the standards set by the Declaration of Helsinki. Clinical data included the patient gender, age, smoking history, limited- or extensive-stage disease and follow-up (Table 1).

### Cell culture and treatment

Human SCLC cell line NCI-H69, NCI-H446, NCI-H69ARwere purchased from the American Type Culture Collection (ATCC, United States of America (USA)) and maintained in RPMI1640 medium contain in l-glutamine with 10% and 20% fetal calf serum respectively in an incubator at 37 °C with 5% CO2. The cisplatin-resistant NCI-H446DDP cell line was obtained by culturing cells in gradually increasing doses of cisplatin up to 2.0 uM after a total of 7 months in our laboratory. The drug-resistant cells were maintained in drug-free medium for at least 2 weeks before any experiment. To inhibit EZH2 activities, cells were treated with 12 μM GSK343 (S7164, Selleck) or 10 μM EI1 (S7611, Selleck) for 36 h.

### RNA isolation, quantitative reverse transcription-polymerase chain reaction (qRT- PCR)

Total RNA was extracted from cell lines and FFPE tissues using TRIzol (Invitrogen), RNeasy FFPE Kit (Qiagen) according to the manufacturer’s instructions. According to Prime Script RT reagent Kit (TIANGEN, Beijing, China), reverse transcription reactions were processed at 42 °C for 15 min, followed by 3 min at 95 °C for cDNA synthesis. Then quantitative real time PCR was performed in an ABI illumina instrument . Primers were designed by Shanghai Sangon Biotech Co Ltd. TUG1 F: 5′ TAGCAGTTCCCCAATCCTTG3′; R: 5′CACAAATTCCCATCAT TCC- C3′; LIMK2b F: 5′ AGGCAGTCACAGACGGATTT3′; R: 5′GAGCTTCCCATCCT- TCTCATAG 3′; GAPDH was used as an endogenous control. The relative gene expression levels of TUG1 were determined using the comparative delta-delta CT method (2^-∆∆Ct^).

### Cell transfection

SCLC cells were transiently transfected with small interfering siRNA or scrambled siRNA negative control (NC) . Following the manufacturer’s protocols, cells were seeded in six-well plates and transfected with siRNA by Lipofectamine 2000 (Invitrogen) when grew to reach about 70% confluence. Three individual TUG1 siRNAs (siTUG1 1*, siTUG1 2*, siTUG1 3*1), EZH2 siRNA (si-EZH2 1*, si-EZH2 2*, si-EZH2 3*) and siNC were designed by GenePharma Inc (Shanghai, China) . The nucleotide sequences of siRNAs for TUG1 and EZH2 were shown in table S1.

The lentiviral particles of shTUG1 (forward, 5′-GATCCGCTTGGCTTCTATTCTG AATCCTTTCAAGAGAAGGATTCAGAATAGAAAGCCAAGCCAAGCTTTTTTG-3′ and reverse, 5′-GCGAACCGAAGATAAGACTTAGGAAAGTTCTCTTCCTAA GTCT TATCTTCGGTTCGAAAAAAC-3′) and LIMK2b-GFP were also designed and purchased from GenePharma Co., Ltd. To generate the lenti-viruses, shRNA plasmids were co-transfected into SCLC cells along with envelope (VSVG) and packaging (pGag/Pol, pRev) plasmids using lipofectamine 2000 (Invitrogen). The viral supernatants were harvested and filtered after 48 h transfection. Cells were infected in the presence of a serum-containing medium supplemented with 8 μg/ml polybrene. Following infection for 48 h, cells were selected with 2.0 μg/ml puromycin (Sigma). Knockdown efficiencies were examined by qRT-PCR.

### Cell counting kit-8 (CCK-8) assay

Cell proliferation and drug resistance were assayed by the Cell Counting Kit-8 (CCK8) assay. For cell proliferation assay, transient transfection cells were seeded in 96-well plates about 5 × 10^3^ cells per well. According to the manufacturer’s protocol, testing cell proliferation every 24 h. For cell drug resistance assay, after transient transfection cells, then treated it with drugs for 24 h. Three chemotherapy drugs [Cisplatin (DDP; Shandong, China), Adriamycin (ADM; Jiangsu, China) Etoposide (VP-16; Jiangsu, China),] were used. After incubation with 10ul of CCK-8 reagent (Beyotime Institute of Biotechnology, shanghai, China) for 2 h or 4 h, the absorbance at 450 nm was measured. The cells incubated without drugs were set at 100% survival and were used to calculate the concentration of each chemotherapeutic drug IC50. The assay was performed in five replicate wells, and three parallel experiments for each sample were conducted.

### Colony formation assay

Collected cells that transduced with shTUG1, both shTUG1 and LIMK2b-GFP or control shRNA and seeded (200 cells/well) in six-well plates. Then, the cells were incubated in an incubator with 5% CO2 at 37 °C. After two weeks later, removed the culture medium, and rinsed cells three times with PBS. Nextly, the cells were fixed with 4% paraformaldehyde, then stained with 0.1% crystal violet. The number of colonies were counted by visual inspection.

### Cell invasion and migration assay

For the invasion assays, 24-well Matrigel invasion chambers

(Corning Incorporated, Corning, NY, USA) was used. After TUG1 knockdown, 3x10^4^ cells were seeded on the upper chamber. To stimulate invasion, the bottom chamber was added 500 μL medium with 20% FBS . After 48 h, cells in the bottom chamber were stained with 0.1% crystal violet, then counted using a microscopy (100 × magnification). Additionally, Wound healing assay was performed for analysis of cell migration. Cells transfected with either shTUG1 or shNC, were seeded on six-well plates, then created an artificial scratch wound with a 100-μl pipette tip. Cells with serum-free medium for a further 24-h incubation. Recovery of the disruption was observed for 0 h, 24 h. Each assay was performed at least three times.

### Flow cytometric analysis

For apoptosis and cell-cycle assay, cells were transfected with siTUG1, then treated with drugs for 24 h or not before collected. Cell apoptosis assay was conducted by using AnnexinV/propidium iodide detection kit (Keygene, Nanjing, China). For cell-cycle assay, cells were collected and fixed in 70% ethanol at 4 °C for 16 h and then stained with propidium iodide.

### Tumor xenograft experiments

This study was conducted according to the institutional guidelines of Guangdong Province and were approved by the institutional guidelines of Guangdong Province and by the Use Committee for Animal Care. Male BALB/c nude mice aged 3–4 weeks were purchased from the Experimental Animal Center of Sun Yat-sen University (Guangzhou, China). Cells were harvested and re-suspended in serum free medium at a concentration of 1 × 10^7^ cells/0.2 ml. Each mouse was inoculated subcutaneously in the right flank with SCLC cells stably transduced with shTUG1 or shControl. Tumor size was monitored every 3 days, and mice were euthanized after 4 weeks. In vivo chemosensitivity assays, the animals were treated with chemotherapeutics or PBS via intraperitoneal injection (7 mg/kg body weight etoposide [once every 2 days] and 3 mg/kg body weight cisplatin [once every 8 days]).

### Western blotting

Equivalent amounts of cell protein lysates were electrophoresed on an 10% SDS-polyacrylamide gel, transferred to a PVDF membrane. Then the membrane was incubated with primary antibodies overnight at 4 °C. Followed incubated by horseradish peroxidase-labeled secondary antibody. Anti-LIMK2b was purchased from Abcam (1:1,000). GAPDH was used as a protein-loading control. The immune complexes were detected by chemiluminescence (ECL).

### RNA immunoprecipitation (RIP) assay

RNA immunoprecipitation was conducted using Magna RIP RNA-Binding Protein Immunoprecipitation Kit (Millipore) following the manufacturer’s protocol.

### Chromatin immunoprecipitation

The ChIP assays were performed according to the Protocol for the fast chromatin immunoprecipitation (ChIP) method [42]. EZH2 antibody was purchased from Abcam. H3 trimethyl Lys 27 antibody was from Millipore. Gene specific primers for LIMK2b are listed in Additional file 5: Table S5. Results were normalized using the internal control IgG. Precipitated chromatin DNA was recovered and analyzed by qPCR.

### Statistical analysis

Statistical analyses were performed using SPSS version 21.0 software. Experimental results are presented using means ± SD. Independent- samples *T* test or one-way ANOVA were used to analyze the possible differences between groups. The association between TUG1 expression and clinical features were analyzed by Pearson Chi-Square test. Survival curves were assessed by Kaplain-Meier analysis. Prognostic factors were analyzed by univariate and multivariate analyses (Cox proportional hazards model). P values < 0.05 was considered statistically significant.

## Additional files

Additional file 1: Figure S1. Relative expression level of TUG1 or EZH2 in H69, H446, H69AR and H446DDP cells transfected with siNC or si-TUG1 or si-EZH2. *, *P* < 0.05; **, *P* < 0.001. (TIF 3849 kb)
Additional file 2: Figure S2. TUG1 promoted migration and invasion of SCLC cells in vitro. (A) Cell migration was quantified by wound healing assay. Cells were imaged immediately (0 h) and 24 h after scratches were created to measure the percentage of wound healed area. Representative images at different time points are shown. (B) Cell morphology graph of invasive cells in H446, H69AR and H446DDP cells after stable transfection of shTUG1 or shControl. Data represent mean ± SD of three independent experiments. (TIF 5658 kb)
Additional file 3: Figure S3. Cell apoptosis and cell cycle were assayed by flow cytometric analysis after H69 and H446 cells were transfected with siTUG1. (TIF 6255 kb)
Additional file 4: Figure S4. Apoptosis of H69AR-siTUG1, H446DDP-siTUG1 cells induced by anticancer drugs was significantly increased compared with controls. (TIF 6733 kb)
Additional file 5: Table S5. qRT-PCR primers. (XLS 20 kb)

## Abbreviations

ADM: Adriamycin
CCK-8: Cell counting kit-8 assay
ChIP: Chromatin immunoprecipitation
DDP: Cisplatin
lncRNAs: Long non-coding RNAs
PRC2: Polycomb repressive complex2
qRT-PCR: Quantitative real-time polymerase chain reaction
RIP: RNA Binding protein immunoprecipitation
SCLC: Small cell lung cancer
VP-16: Etoposide
