# Correction: Long non-coding RNA TUG1 is involved in cell growth and chemoresistance of small cell lung cancer by regulating LIMK2b via EZH2

**DOI:** 10.1186/s12943-024-02026-7

**Published:** 2024-05-25

**Authors:** Yuchun Niu, Feng Ma, Weimei Huang, Shun Fang, Man Li, Ting Wei, Linlang Guo

**Affiliations:** 1 Department of Pathology Zhujiang Hospital, Southern Medical University, 253 Gongye Road, Guangzhou, 510282 People’s Republic of China; 2 Department of Oncology, The First Affiliated Hospital of Hebei NorthUniversity, Zhangjiakou, China; 3 Department of Oncology, Zhujiang Hospital, Southern Medical University, Guangzhou, China

**Correction:** ***Mol Cancer*16, 5 (2017)**

10.1186/s12943-016-0575-6

Following publication of the original article [1], the authors noticed that two images (Figs. 2E and 5C) in the plate clone experiment section were accidentally duplicated due to personal negligence.

They fully understand that this mistake may have caused inconvenience to the readers and raised doubts about the rigor of their research. The authors deeply apologize for this oversight and have immediately taken corrective measures.

The authors hereby confirm that this error was solely caused by carelessness and does not involve any form of academic misconduct. They have sufficient original experimental images as evidence to prove that the duplication was the result of mistakenly placing the wrong images. Although this error concerns the results of the proliferation experiment, the paper also used the CCK8 proliferation assay to validate their findings. In addition, their article also proved the effect on cell proliferation through in vivo experiments, further confirming their conclusion. Therefore, this error does not affect their conclusions regarding the impact on cell proliferation or the main findings of the paper. The correct Fig. 2 is given below.

**Fig. 2:**
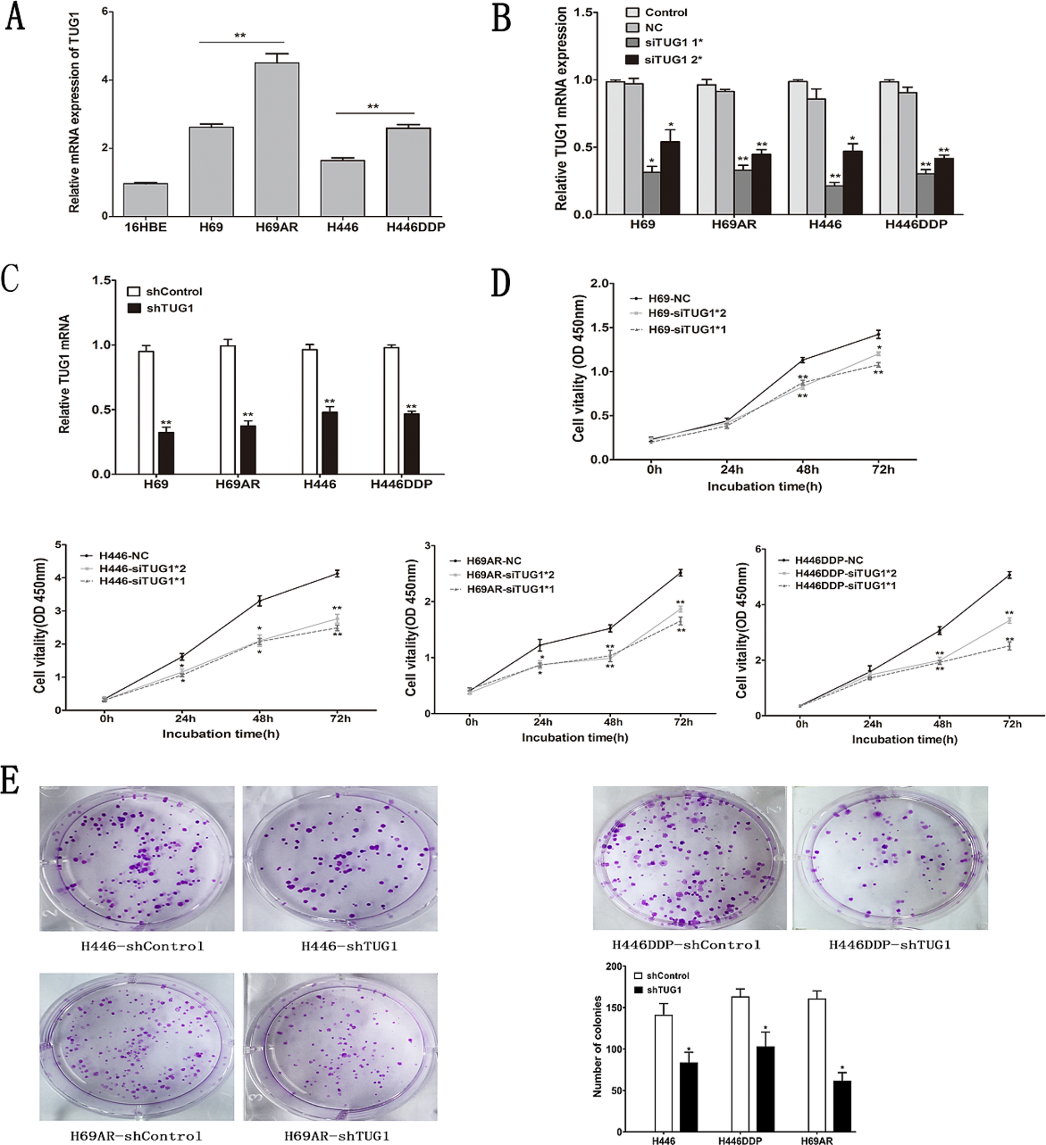
TUG1 was up-regulated in SCLC cell lines and TUG1 knockdown inhibited cell proliferation in vitro. **a** The expression of TUG1 was assessed in SCLC cell lines compared with the normal bronchial epithelial cell line (16HBE) by qRT-PCR. **b c** Inhibition of TUG1 by transfection of TUG1 siRNAs or sh RNA in H69、H69AR、H446、H446DDP cells. **d** CCK-8 proliferation assays were used to determine the cell viability for siTUG1 transfected SCLC cells. Experiments were performed in triplicate. **e** Colony formation assays were performed to determine the proliferation of shTUG1 transfected H446, H446DDP and H69AR cells. Representative photographs are shown, and the numbers of colonies were counted. *, *P* < 0.05; **, *P* < 0.001

## References

[1] Niu Y, Ma F, Huang W (2017). Long non-coding RNA TUG1 is involved in cell growth and chemoresistance of small cell lung cancer by regulating LIMK2b via EZH2. Mol Cancer.

