# Supplementary figures and images for: Long non-coding RNA TUG1 is involved in cell growth and chemoresistance of small cell lung cancer by regulating LIMK2b via EZH2
Source: Mol Cancer. 2017 Jan 9;16:5. doi: 10.1186/s12943-016-0575-6 (PMC5223434; doi:10.1186/s12943-016-0575-6)

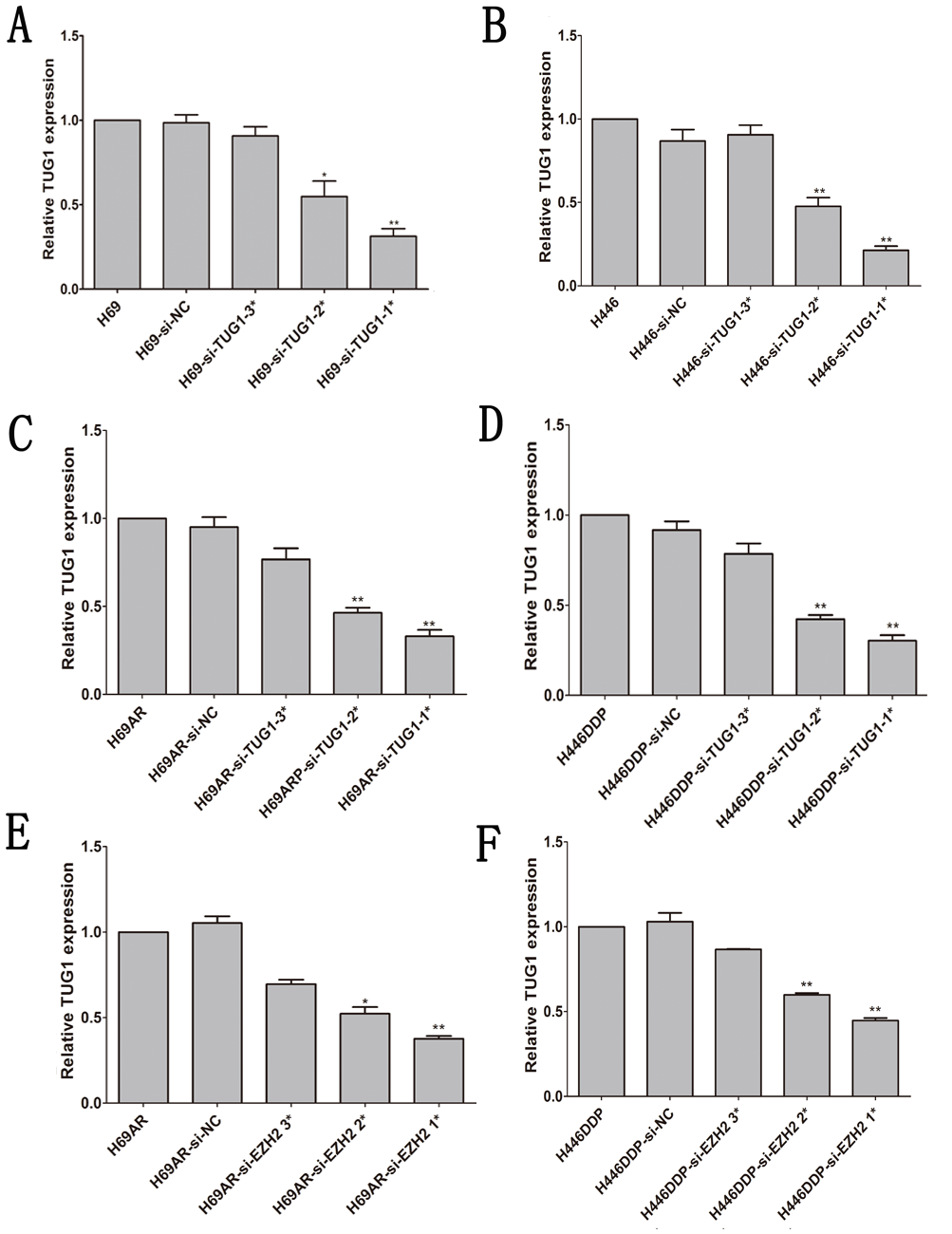

Supplement: Additional file 1: Figure S1. — Relative expression level of TUG1 or EZH2 in H69, H446, H69AR and H446DDP cells transfected with siNC or si-TUG1 or si-EZH2. *, P < 0.05; **, P < 0.001. (TIF 3849 kb) [file 12943_2016_575_MOESM1_ESM.tif]

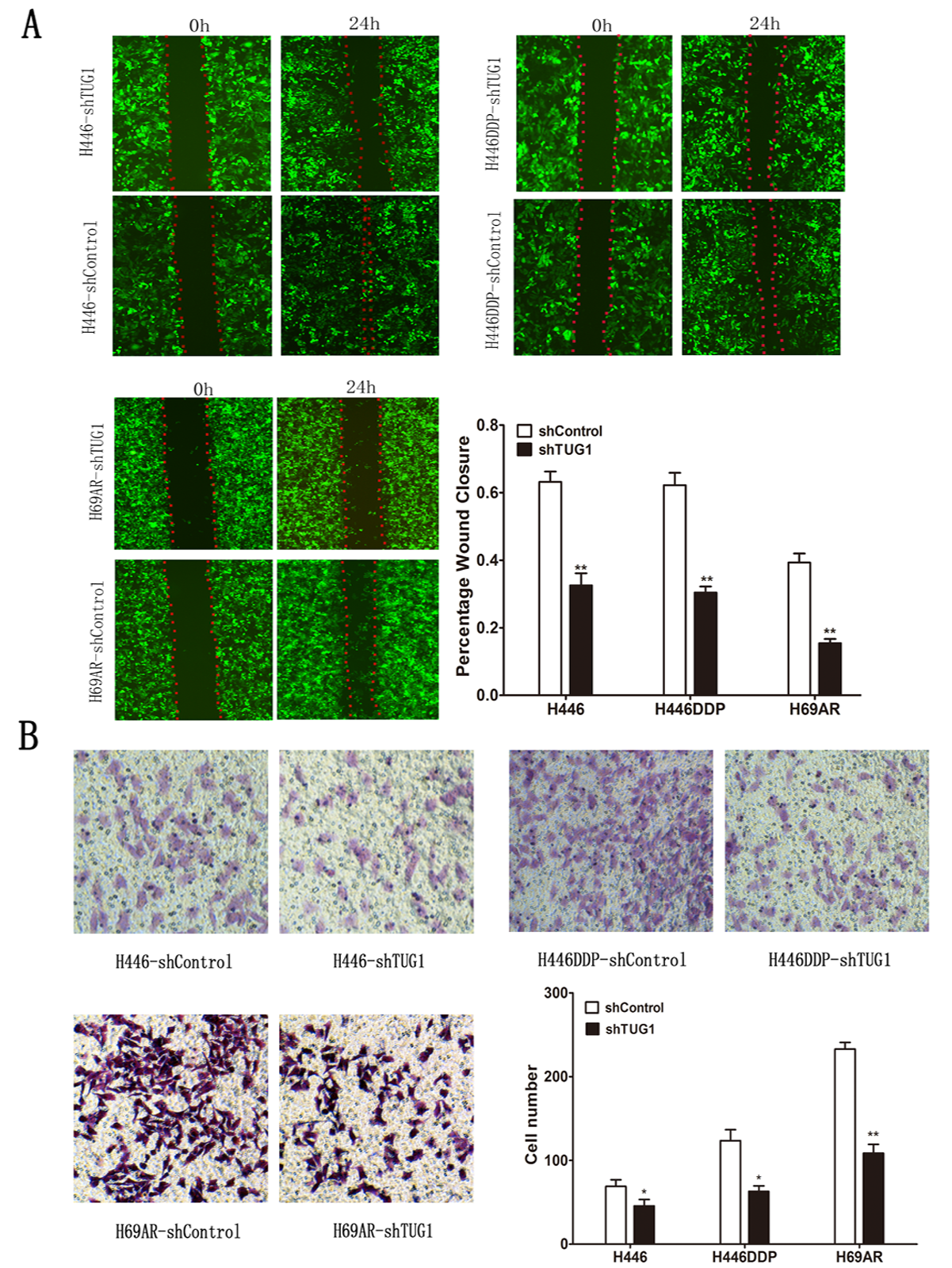

Supplement: Additional file 2: Figure S2. — TUG1 promoted migration and invasion of SCLC cells in vitro. (A) Cell migration was quantified by wound healing assay. Cells were imaged immediately (0 h) and 24 h after scratches were created to measure the percentage of wound healed area. Representative images at different time points are shown. (B) Cell morphology graph of invasive cells in H446, H69AR and H446DDP cells after stable transfection of shTUG1 or shControl. Data represent mean ± SD of three independent experiments. (TIF 5658 kb) [file 12943_2016_575_MOESM2_ESM.tif]

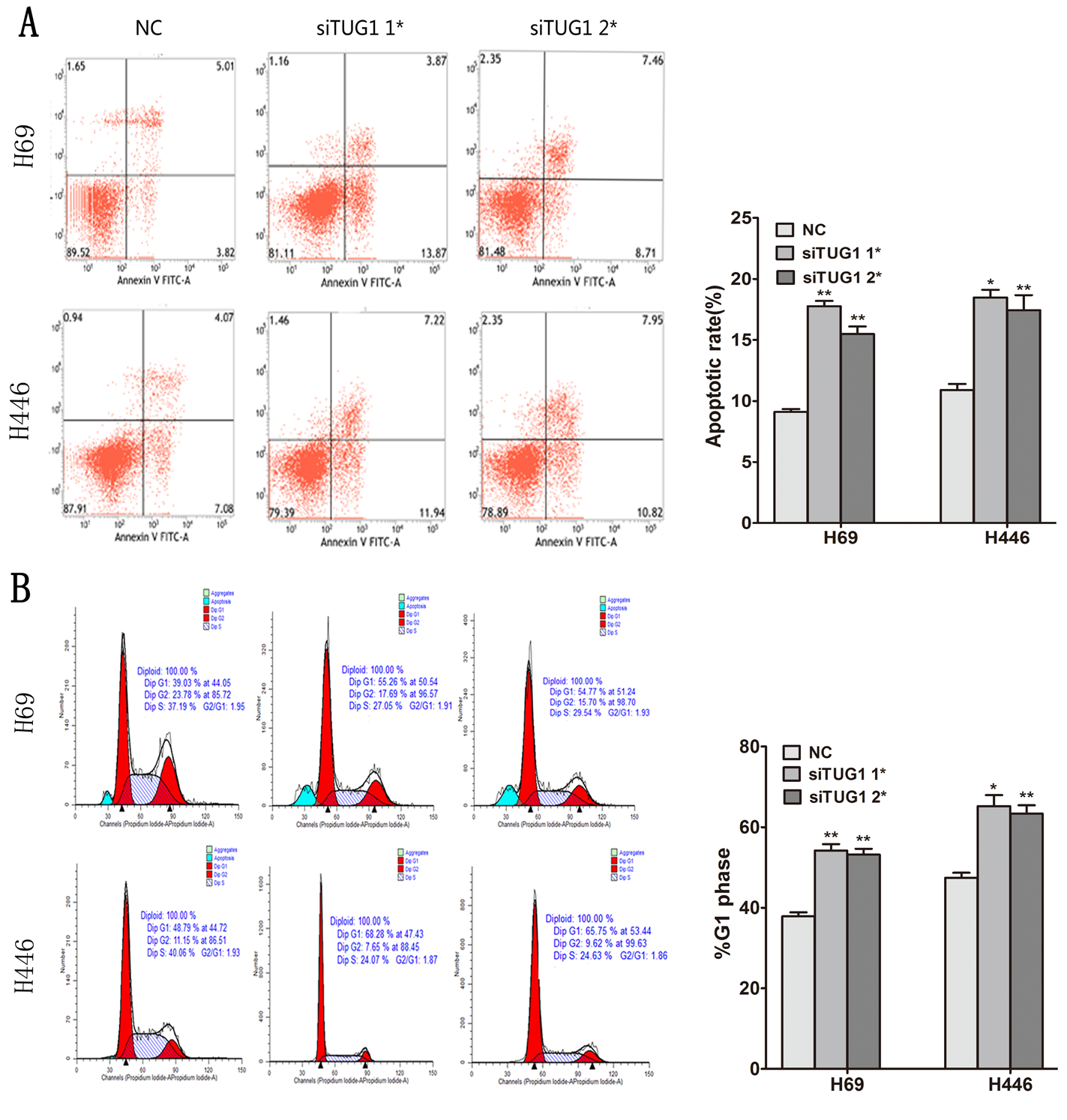

Supplement: Additional file 3: Figure S3. — Cell apoptosis and cell cycle were assayed by flow cytometric analysis after H69 and H446 cells were transfected with siTUG1. (TIF 6255 kb) [file 12943_2016_575_MOESM3_ESM.tif]

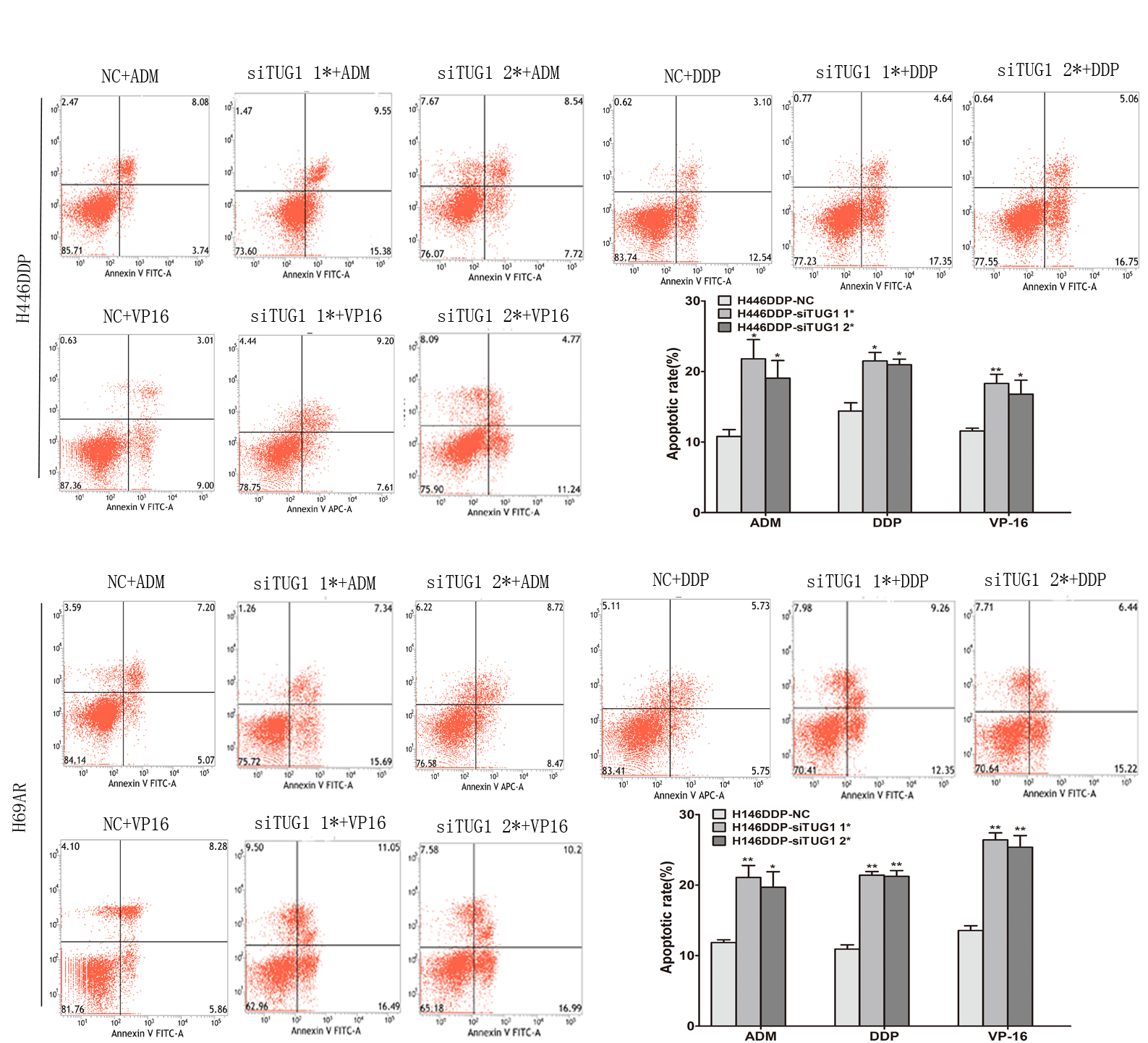

Supplement: Additional file 4: Figure S4. — Apoptosis of H69AR-siTUG1, H446DDP-siTUG1 cells induced by anticancer drugs was significantly increased compared with controls. (TIF 6733 kb) [file 12943_2016_575_MOESM4_ESM.tif]
